# Supplementary figures and images for: The alternative reality of plant mitochondrial DNA: One ring does not rule them all
Source: PLoS Genet. 2019 Aug 30;15(8):e1008373. doi: 10.1371/journal.pgen.1008373 (PMC6742443; doi:10.1371/journal.pgen.1008373)

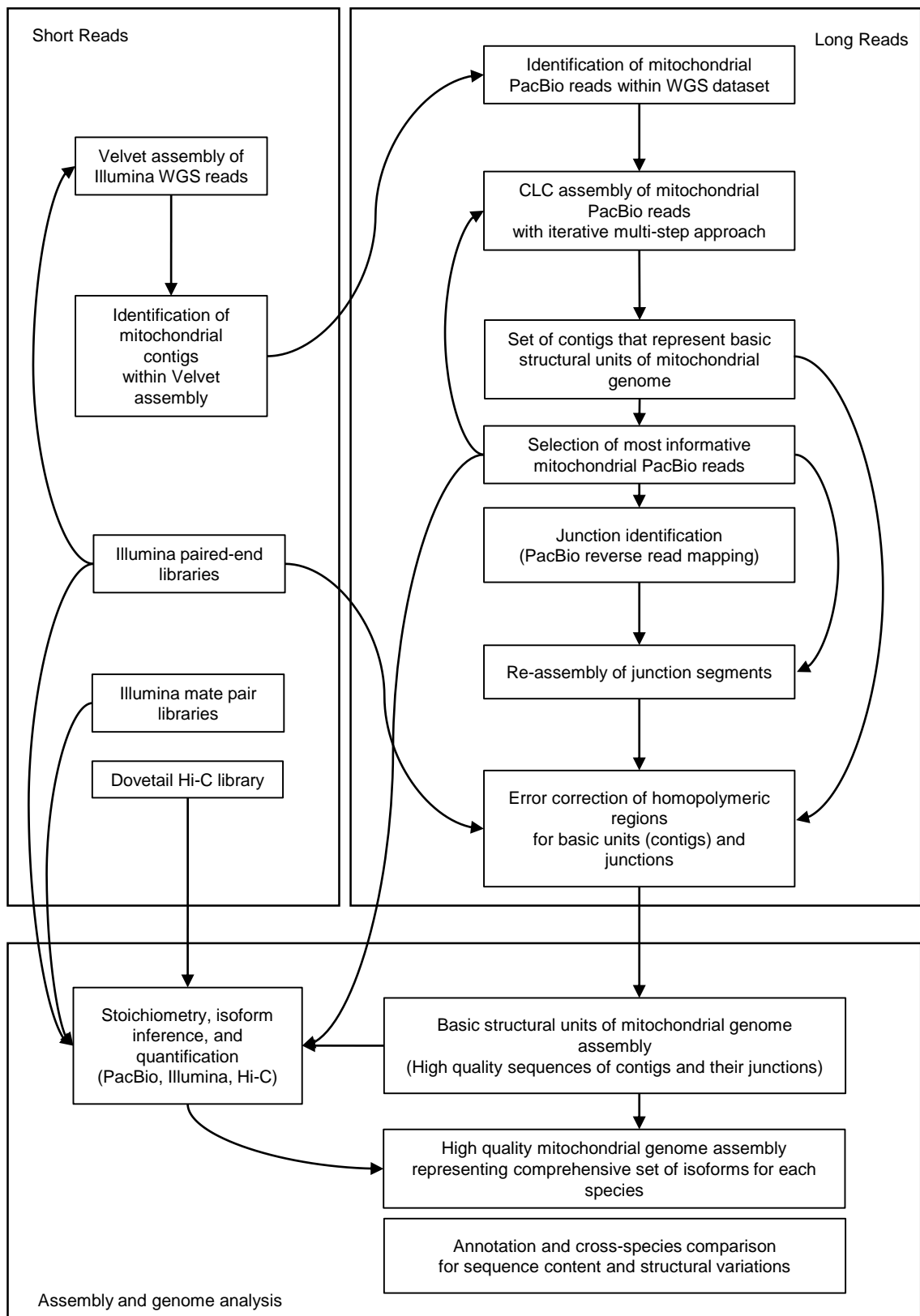

Supplement: S1 Fig — The flowchart summarizes all of the key steps of the Lactuca mitochondrial genome project along with the source and type of raw data used in each iteration of the assembly and analysis. (PDF) [file pgen.1008373.s001.pdf]

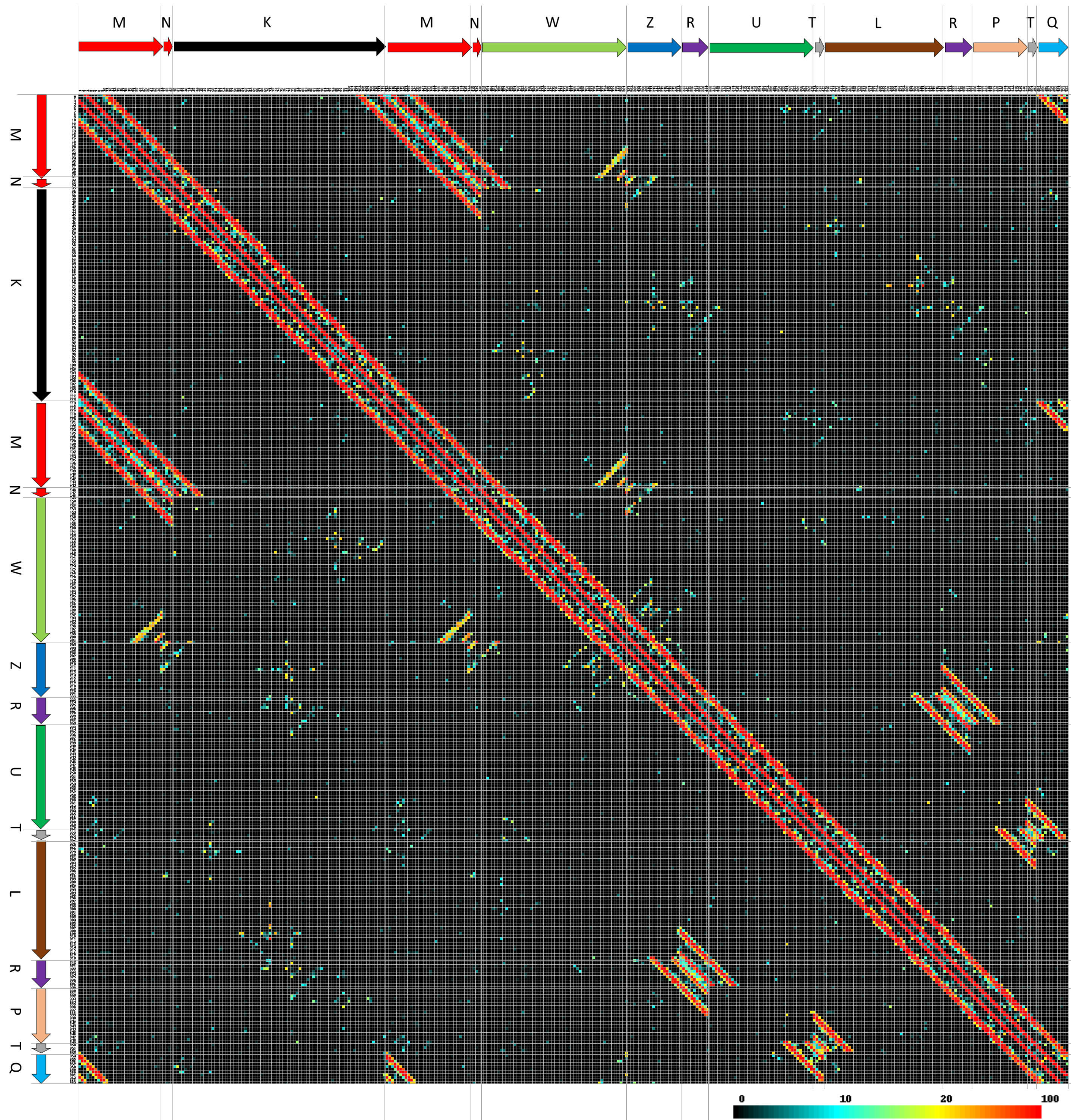

S4A Figure

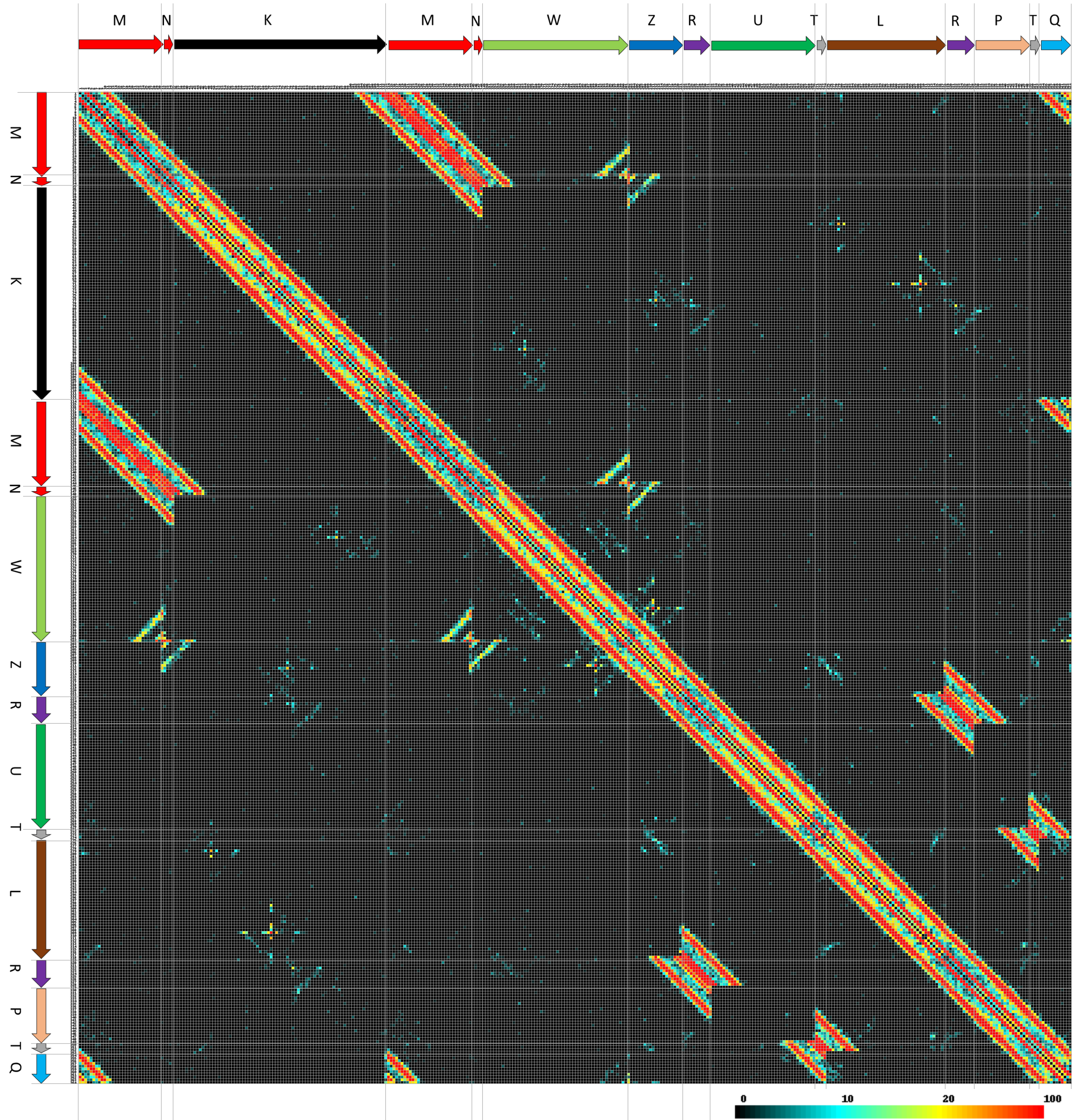

S4B Figure

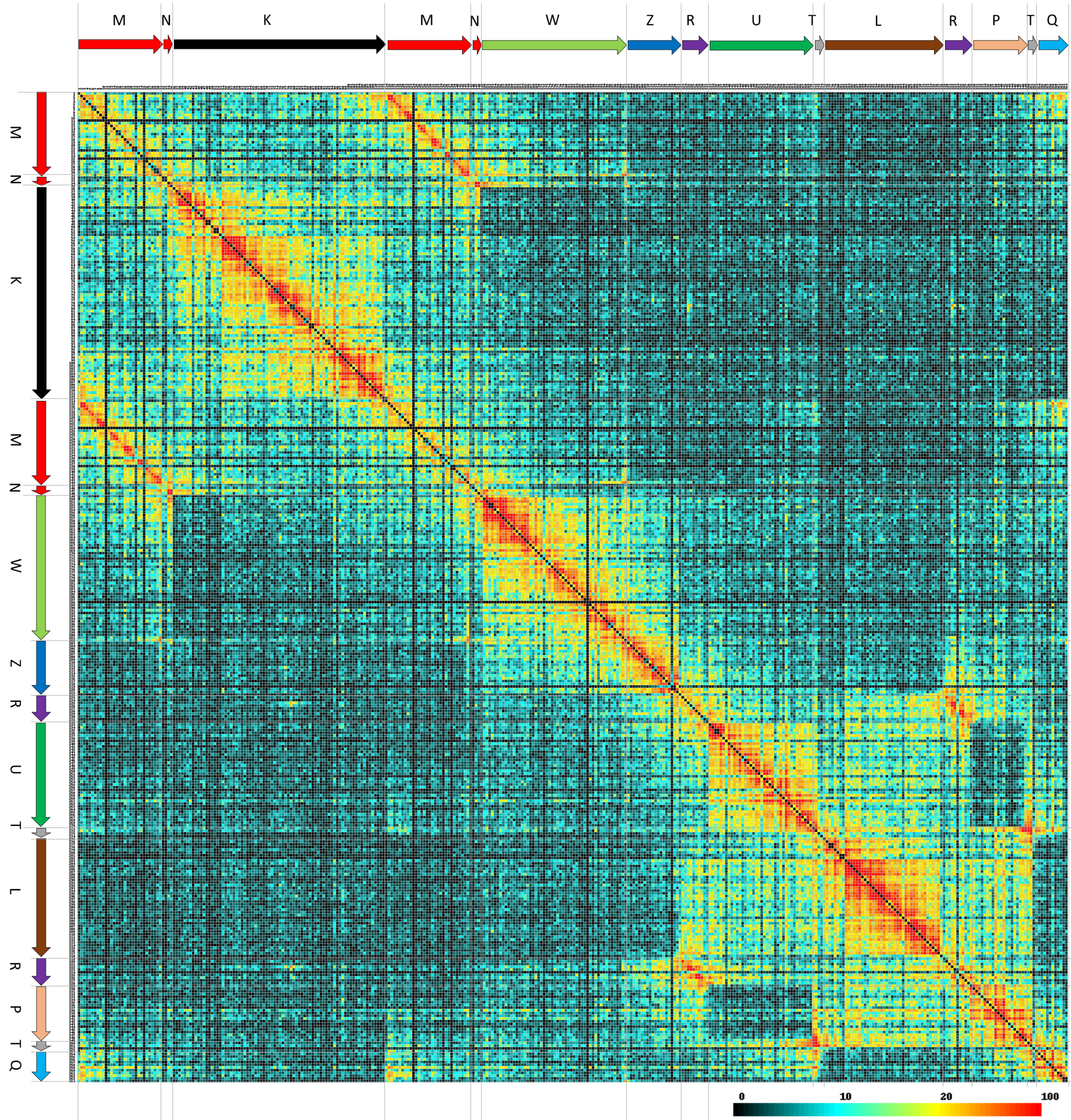

S4C Figure

Supplement: S4 Fig — (PDF) [file pgen.1008373.s004.pdf]

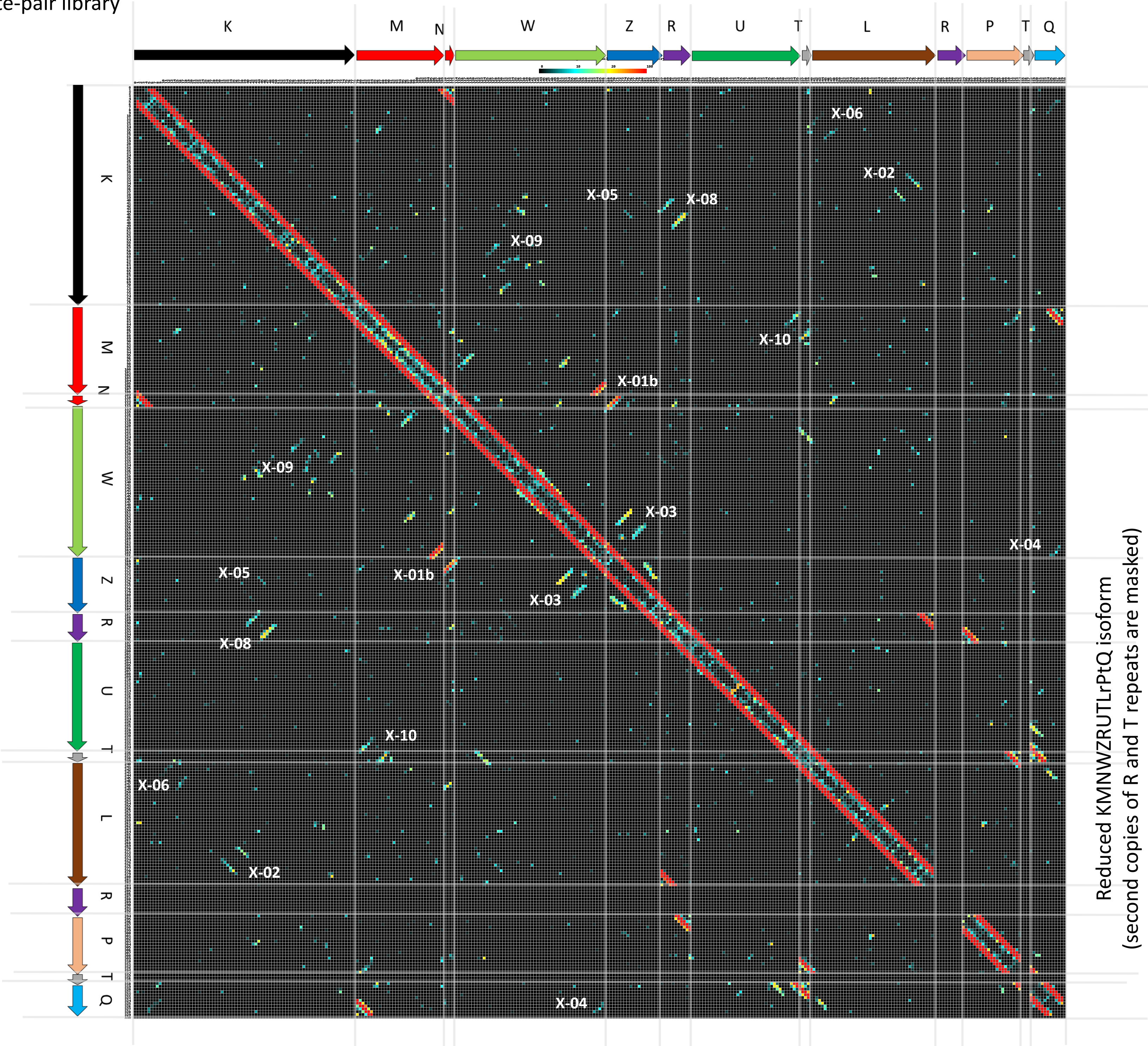

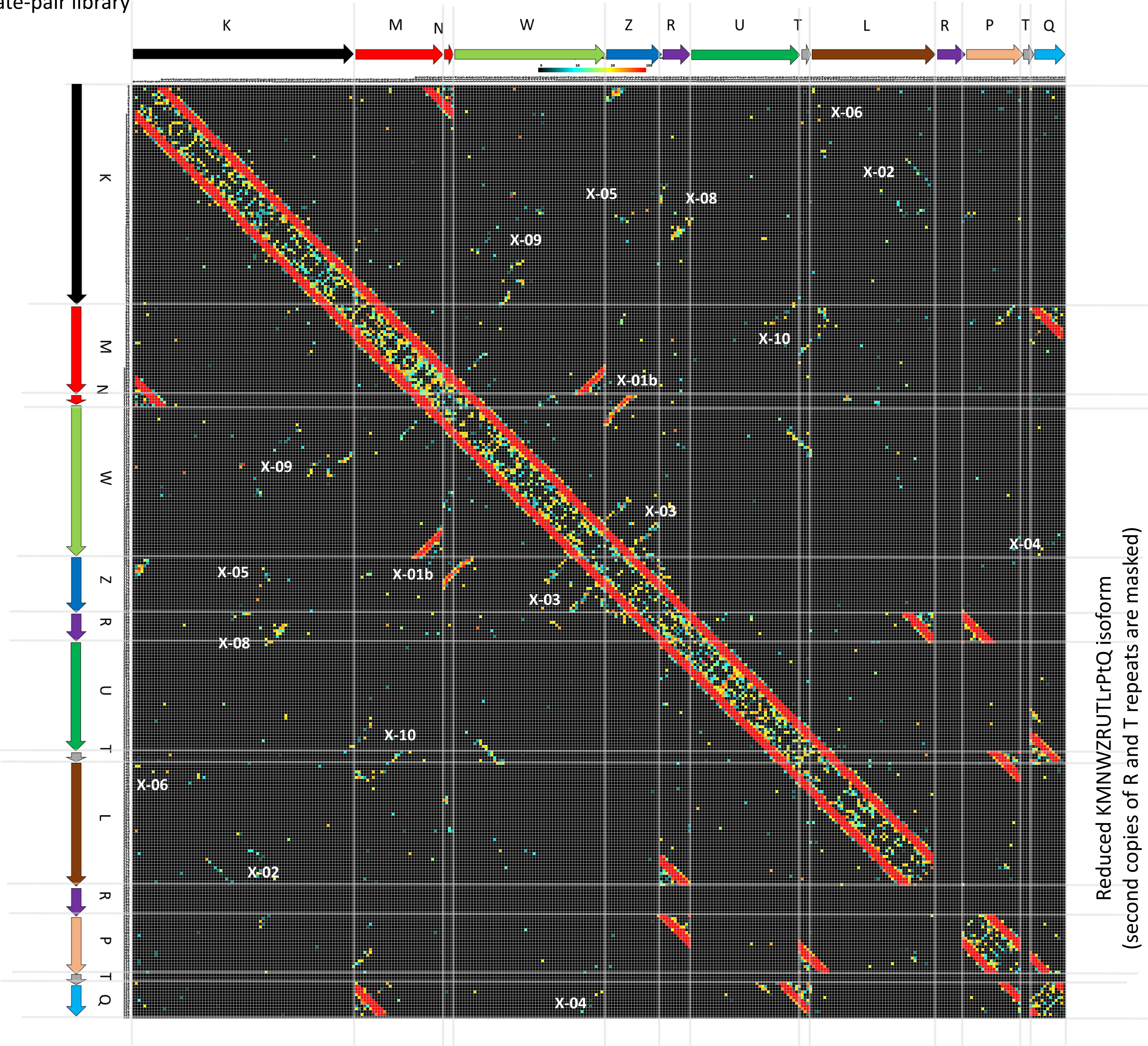

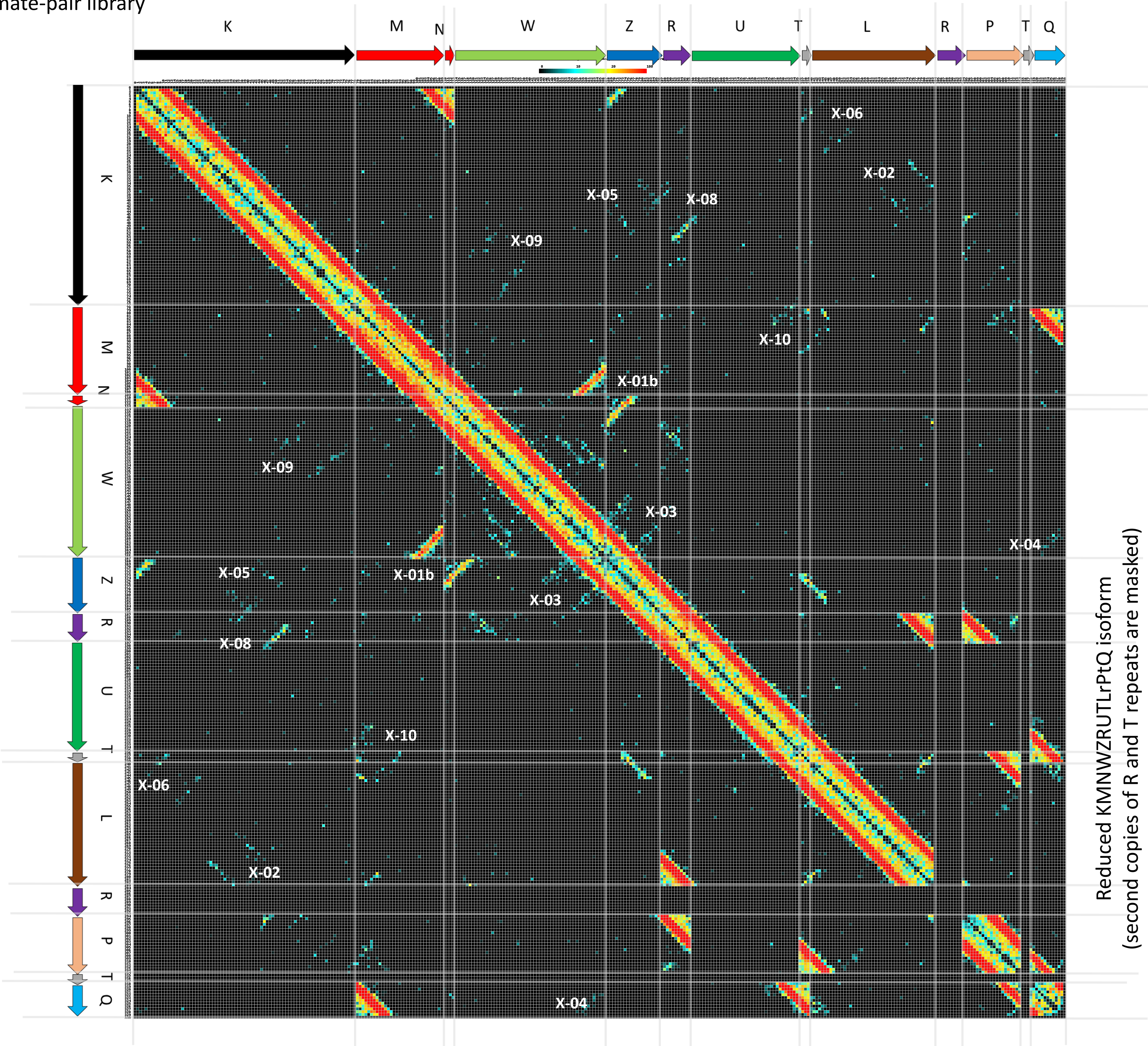

S5C Figure

### X-01b and X-03 using the 5 kb mate-pair *L. sativa* library

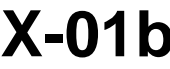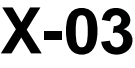

## S5D Figure

Supplement: S5 Fig — Plots of distances between read-pairs in 1 kb bins for L. sativa mate pair libraries of 5 kb (A) and 10 kb insert sizes (B), and L. serriola 10 kb (C). Only unique read mappings were selected from all available reads for the analysis and data interpretation. S5D Fig: Example of numerical values for detection of recombination between short repeats X-01b and X-03 using the 5 kb mate-pair L. sativa library (see S5A Fig for the complete 2D plot). Each cell is a 1 kb bin across the mitochondrial genome isoform α. Values within each cell give the number of times mate-pair reads mapped within the same bin. The majority of mate-pair reads are mapped and equally distributed over the main diagonal and within large repeats (R or T). Rare recombination events between short repeats generate distinct shapes (shown as X-01b and X-03) that are located away from the main diagonal. The values reflect the frequency of recombination. Thus, for repeat X-01b, it could be estimated that corresponding recombination frequency is ~10% and ~1% for repeat X-03. (PDF) [file pgen.1008373.s005.pdf]

# Mitochondrial DNA branched linear structures

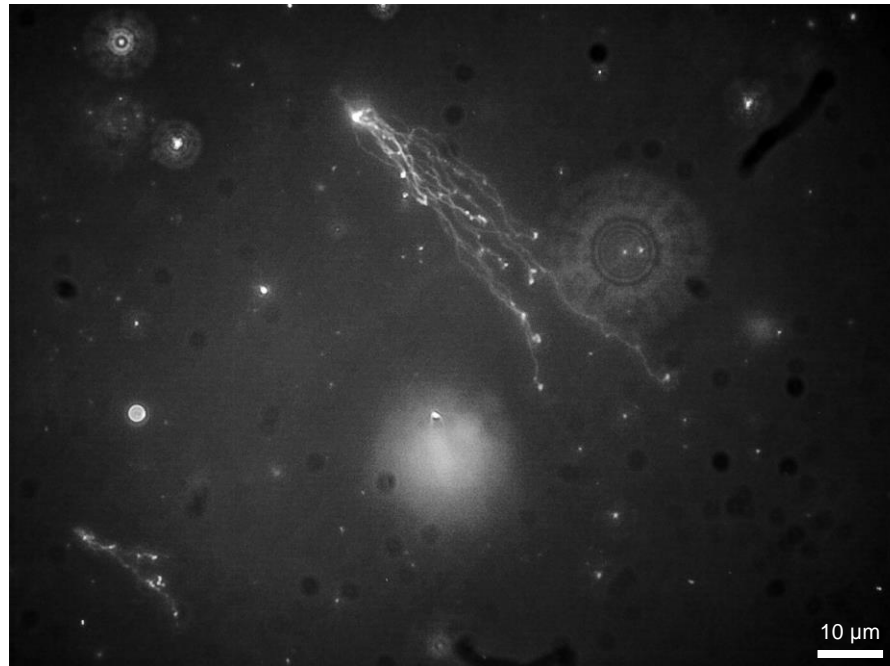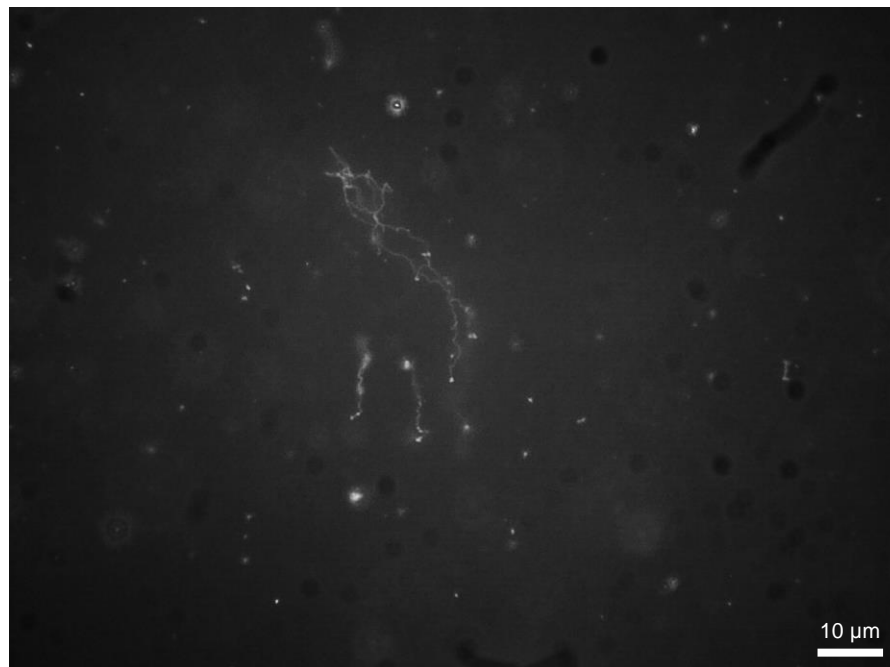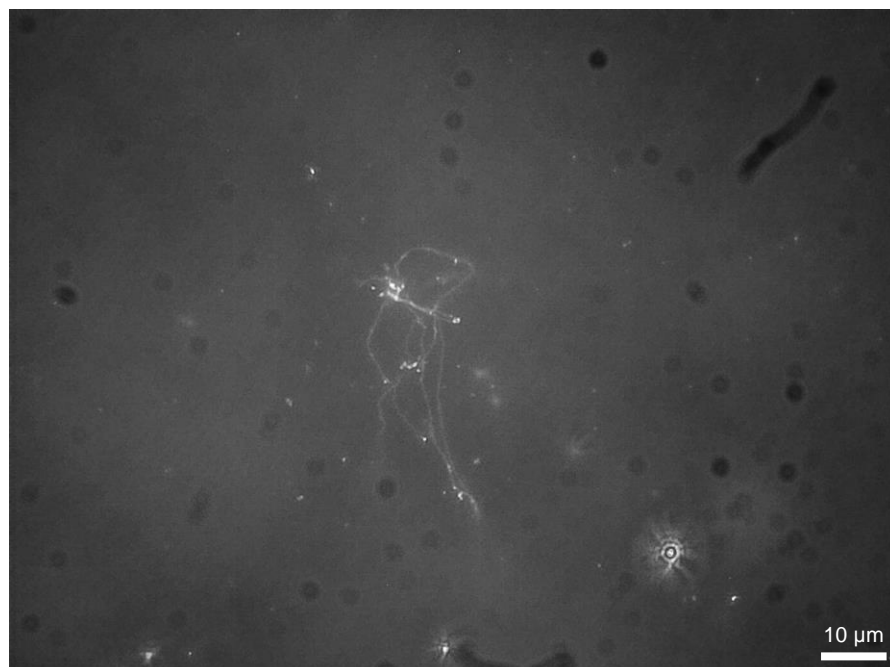

Supplement: S7 Fig — Additional examples of molecules scored in Fig 7G. (PDF) [file pgen.1008373.s007.pdf]
