# Supplementary material for: The alternative reality of plant mitochondrial DNA: One ring does not rule them all
Source: PLoS Genet. 2019 Aug 30;15(8):e1008373. doi: 10.1371/journal.pgen.1008373 (PMC6742443; doi:10.1371/journal.pgen.1008373)
Supplement: S2 Fig — A tiling library of overlapping fragments (similar to that used for reverse read mapping) was used to estimate coverage for each fragment (see Contig Stoichiometry in Materials and methods). The Y axis shows coverage; the X axis displays the coordinates of the primary structural units. Note the elevated coverage in the middle portion in the majority of basic units. Stoichiometry values for each primary structural unit were used for the mitochondrial genome isoform modeling. (PDF) [file pgen.1008373.s002.pdf]

L. sativa mitochondrial genome units coverage by PacBio reads

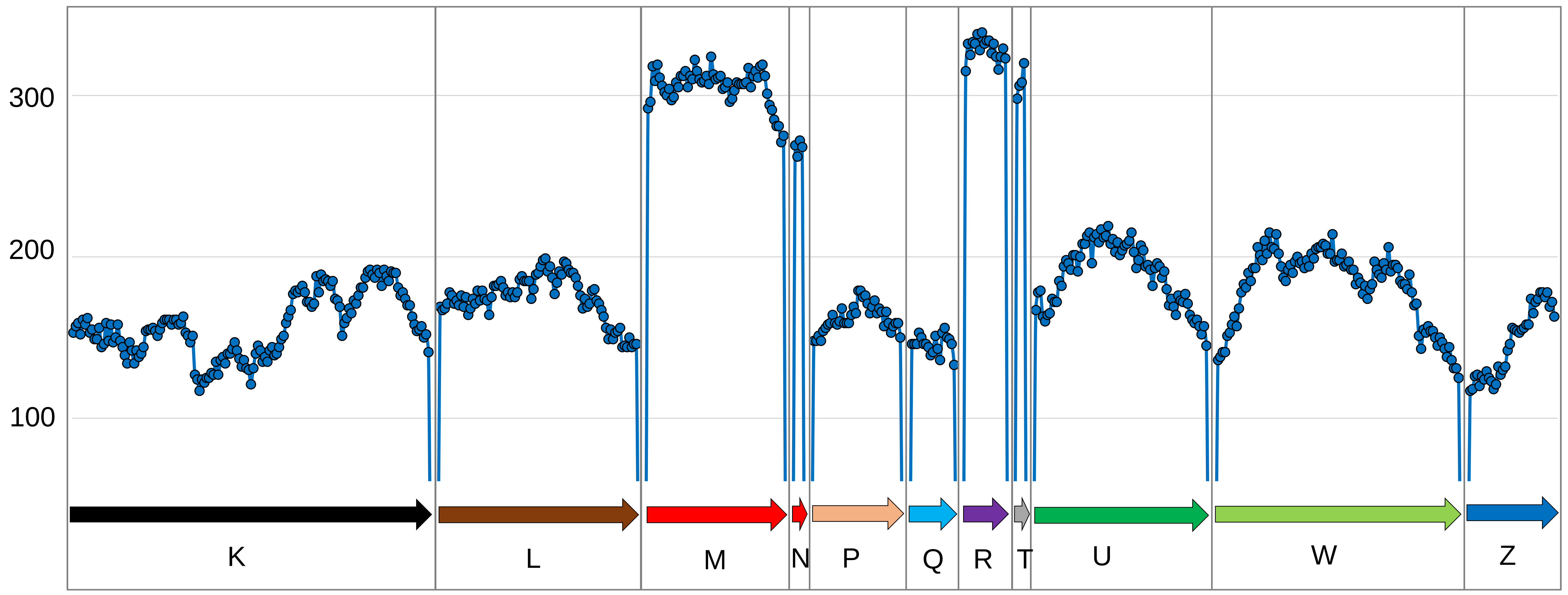

L. saligna mitochondrial genome units coverage by PacBio reads

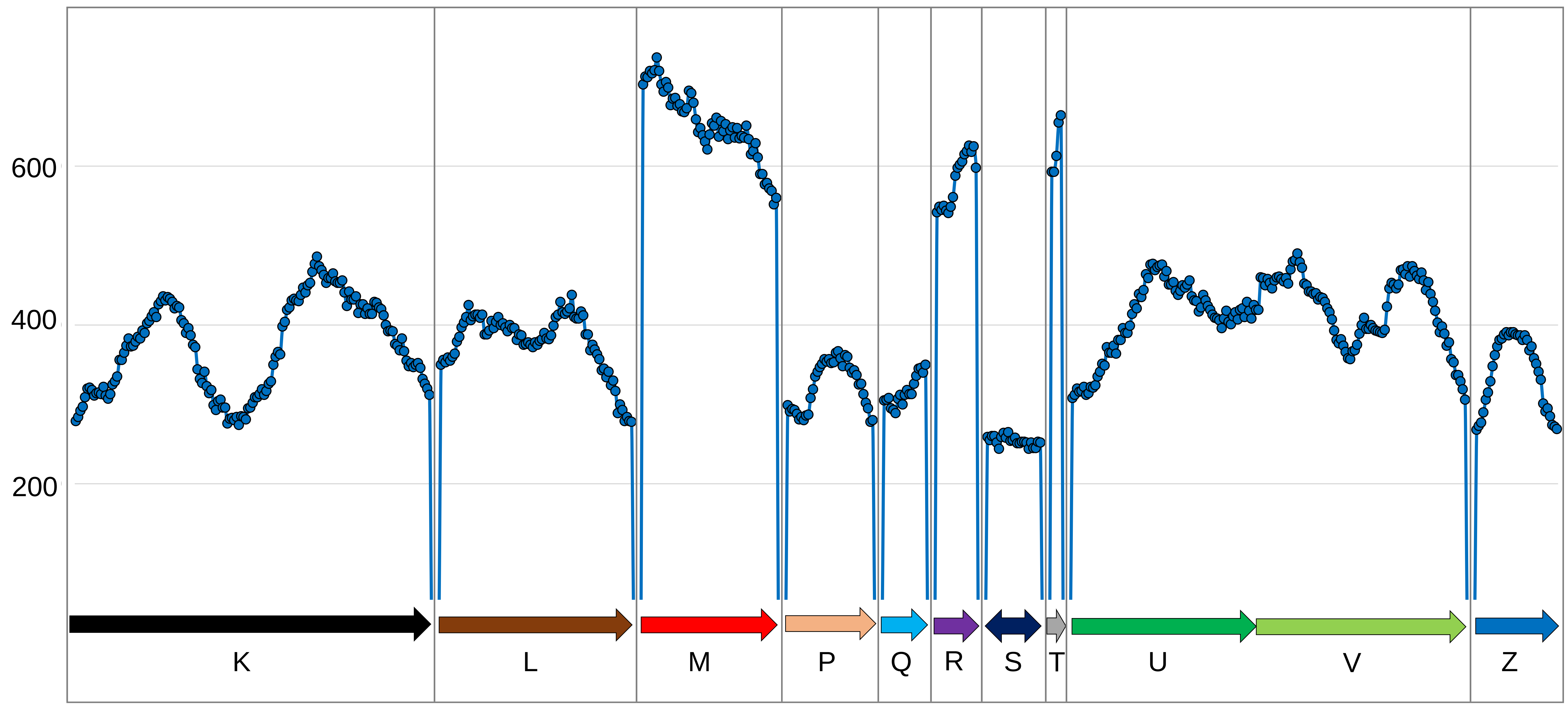

X axis - 2000 bp long tiling overlapping segments across mitochondrial genome units
Y axis - number of PacBio read alignments to the tiling queries

S2 Figure
