# Supplementary material for: The alternative reality of plant mitochondrial DNA: One ring does not rule them all
Source: PLoS Genet. 2019 Aug 30;15(8):e1008373. doi: 10.1371/journal.pgen.1008373 (PMC6742443; doi:10.1371/journal.pgen.1008373)
Supplement: S3 Fig — The set (library) of all identified junctions (2 kb each, see Materials and methods, Mitochondrial Contig Stoichiometry) was analyzed for the fraction of uninterrupted alignments within a pool of most informative PacBio reads for L. sativa (A) and L. saligna (B). All BLAST-N alignments are plotted on the X axis and sorted according to alignment lengths. The length of each alignment is shown on the Y axis. Breakpoints (transitions between uninterrupted alignments longer than 1.8 kb) are an indication of the abundance of each particular junction in a pool of PacBio reads. This value (number of uninterrupted alignments) reflects the proportion of any particular junction within isoforms of a mitochondrial genome. (PDF) [file pgen.1008373.s003.pdf]

Quantification of *L.sativa* mitochondrial genome junctions

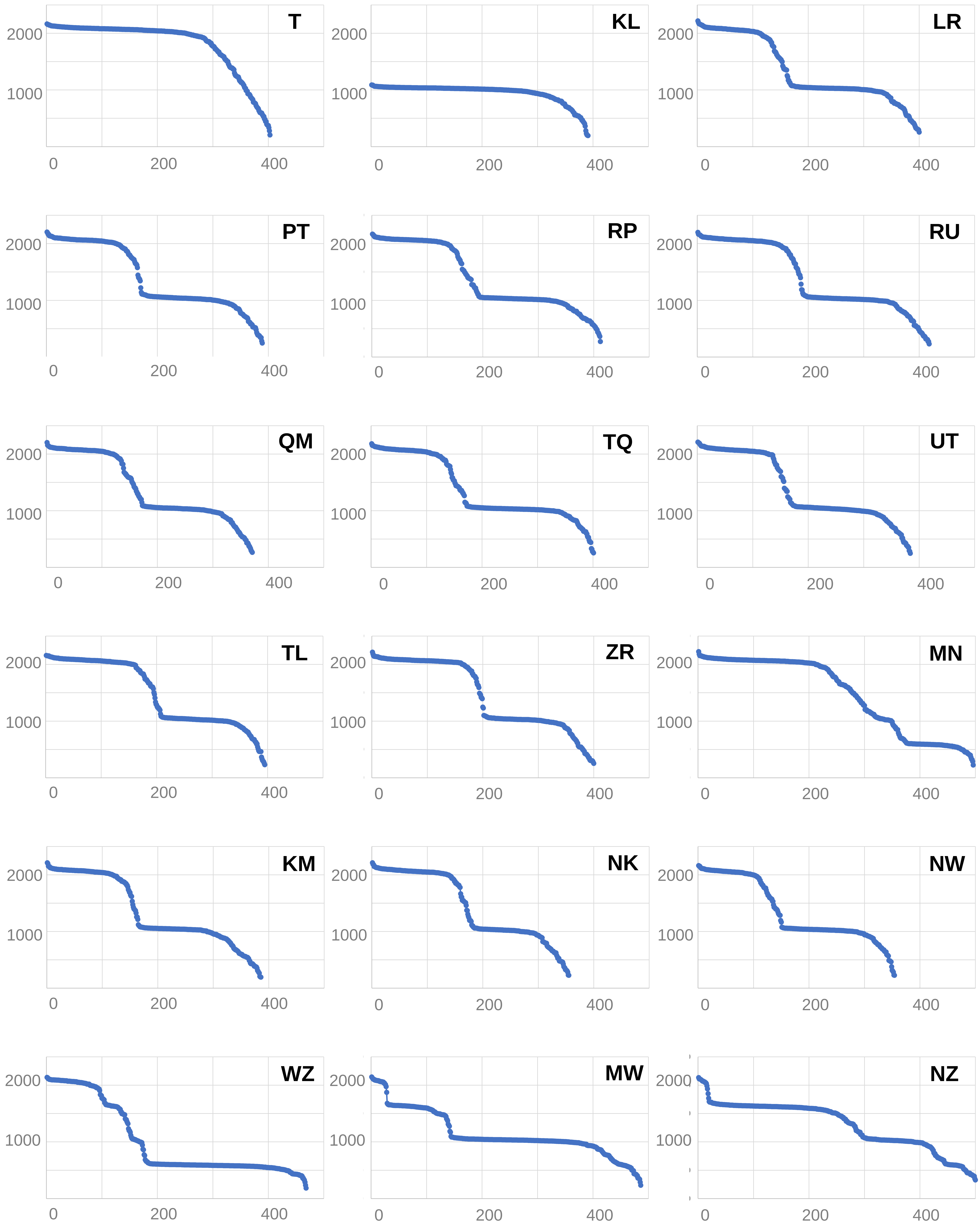

X axis - PacBio reads aligned to 2000 bp junction query and sorted by alignment length
Y axis - alignment length to 2000 bp junction query

S3A Figure

Quantification of *L.saligna* mitochondrial genome junctions

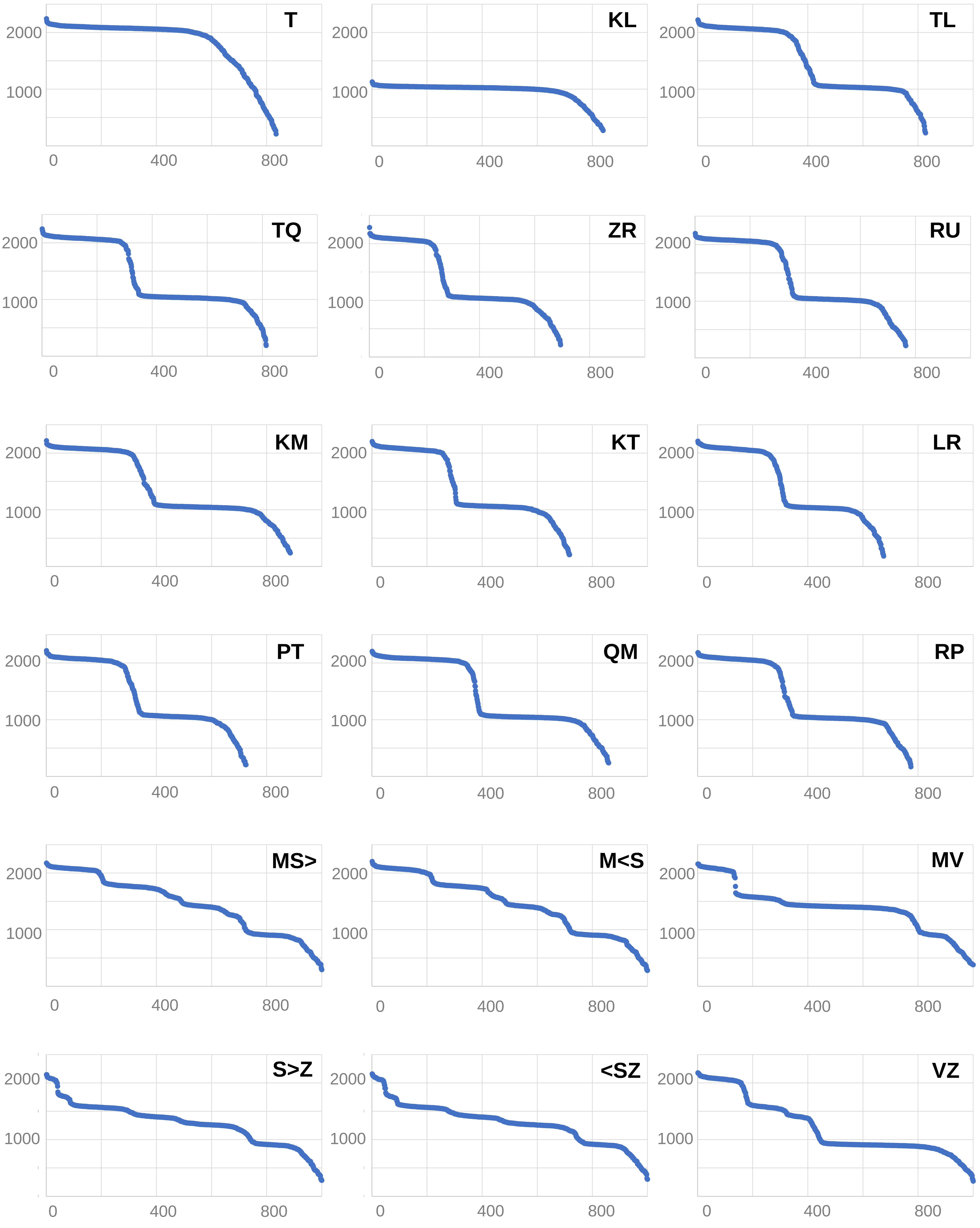

X axis - PacBio reads aligned to 2000 bp junction query and sorted by alignment length
Y axis - alignment length to 2000 bp junction query

S3B Figure
