# Supplementary material for: The alternative reality of plant mitochondrial DNA: One ring does not rule them all
Source: PLoS Genet. 2019 Aug 30;15(8):e1008373. doi: 10.1371/journal.pgen.1008373 (PMC6742443; doi:10.1371/journal.pgen.1008373)
Supplement: S8 Fig — Fourteen contigs derived after CLC assembly of the selected PacBio reads (SRX2719625) were ordered using the published linear assembly accession MH717173 along with autonomous element X (accession MH717174, contig 10) using additional information about the sequential order of contigs based on read-through data (reverse read mapping) and long distance analysis with mate-pair reads. Long distance analysis with mate-pair reads clearly demonstrated that the organization of the Leucaena trichandra mitochondrial genome can be represented in the form of a cyclic graph. All genome segments are linked to each other through several repeats with potential complex rearrangements. Former autonomous element X was placed between a set of repeats that have different segmentation in other parts of the genome. (PDF) [file pgen.1008373.s008.pdf]

Reanalysis of the mitochondrial genome of *Leucaena trichandra* with PacBio and Illumina mate-pair reads.

Contig ID	Copy#	Length
1	1	186,341
2	1	172,341
3	1	63,894
4	1	56,950
5	1	29,925
6	1	32,568
7	2	43,847
8	2	31,854
9	1	15,237
10	1	7,954
11	3	7,686
12	2	6,421
13	3	1,653
14	2	12,500

Fourteen contigs derived after CLC assembly of the selected PacBio reads (SRX2719625) were ordered using the published linear assembly accession MH717173 along with autonomous element X (accession MH717174, contig 10) using additional information about the sequential order of contigs based on read-through data (reverse read mapping) and long distance analysis with mate-pair reads. Long distance analysis with mate-pair reads clearly demonstrated that the organization of *Leucaena trichandra* mitochondrial genome can by represented in the form of a cyclic graph. All genome segments are linked to each other through several repeats with potential complex rearrangements. Former autonomous element X was placed between a set of repeats that have different segmentation in other parts of the genome.

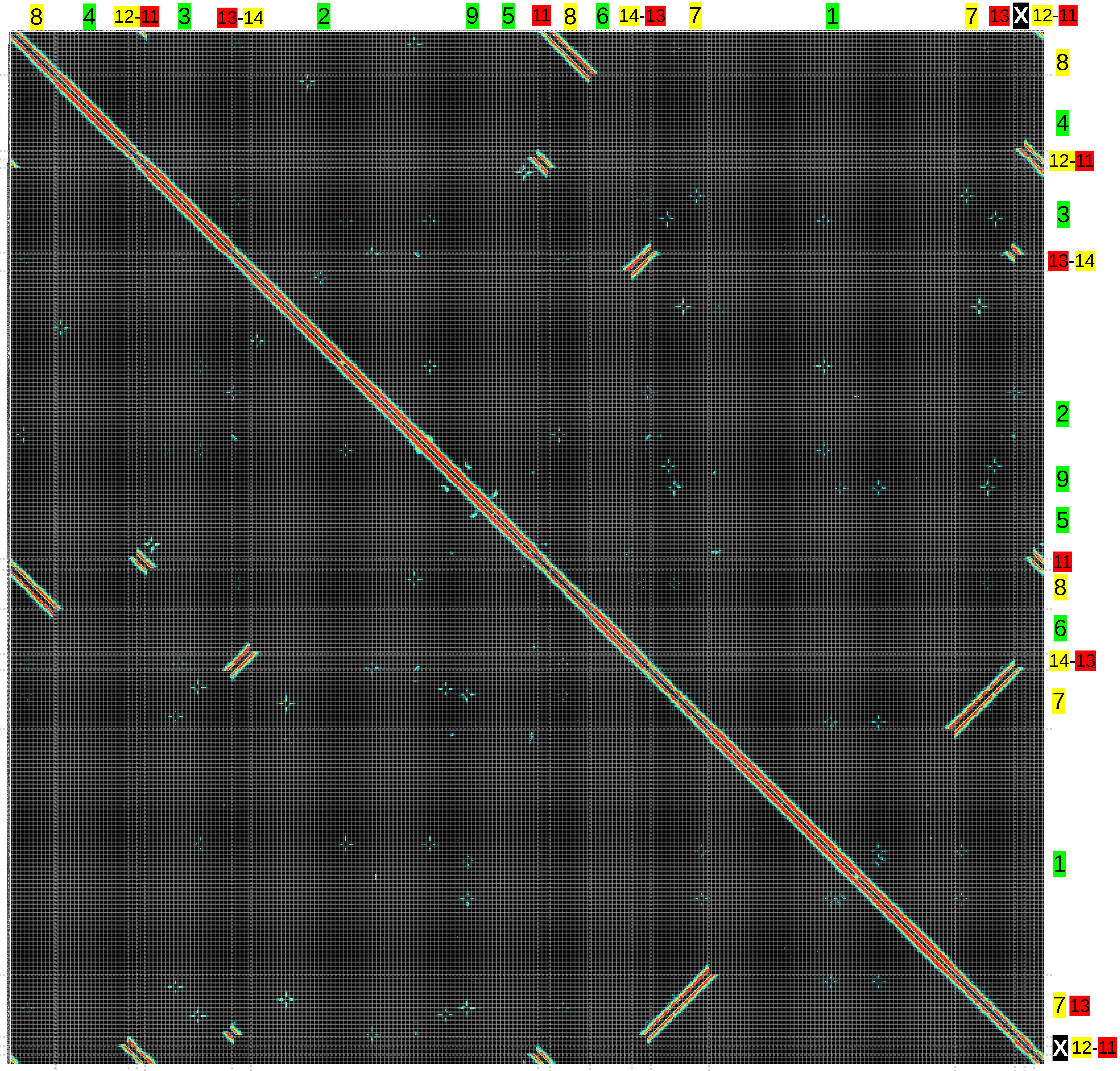

S8 Figure
