# Supplementary material for: The alternative reality of plant mitochondrial DNA: One ring does not rule them all
Source: PLoS Genet. 2019 Aug 30;15(8):e1008373. doi: 10.1371/journal.pgen.1008373 (PMC6742443; doi:10.1371/journal.pgen.1008373)
Supplement: S9 Fig — Explanation and data interpretation of the reverse read mapping approach. Panel A: Scheme of overlapping tiling fragments for primary structural units and reverse mapping protocol outline. Tiling fragments were used as queries in BLAST-N searches versus a database of mitochondrial PacBio reads. Results of BLAST-N were parsed and exported into an MS Excel table as shown in (B). Visual inspection of the distribution of primary structural units over long PacBio reads in an MS Excel table with subsequent search queries on text files provided information about the sequential order of primary structural units within the PacBio reads. This ultimately led to the identification of secondary building blocks (see example of distinct L-T-P and L-T-U blocks detected on a set of PacBio reads). (PDF) [file pgen.1008373.s009.pdf]

A Scheme of overlapping tiling fragments for primary structural units

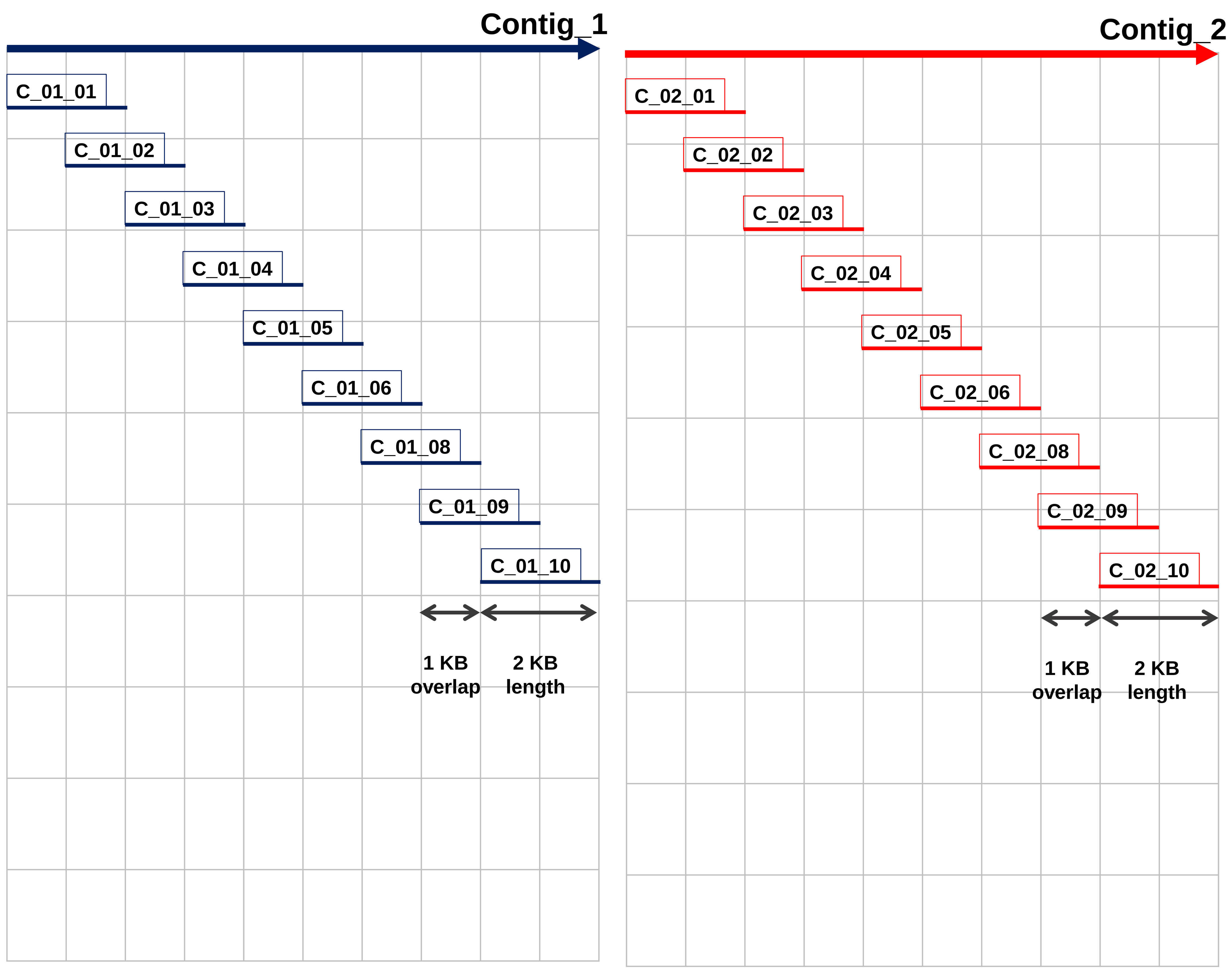

B Reverse Read Mapping coordinates and inference of secondary building blocks

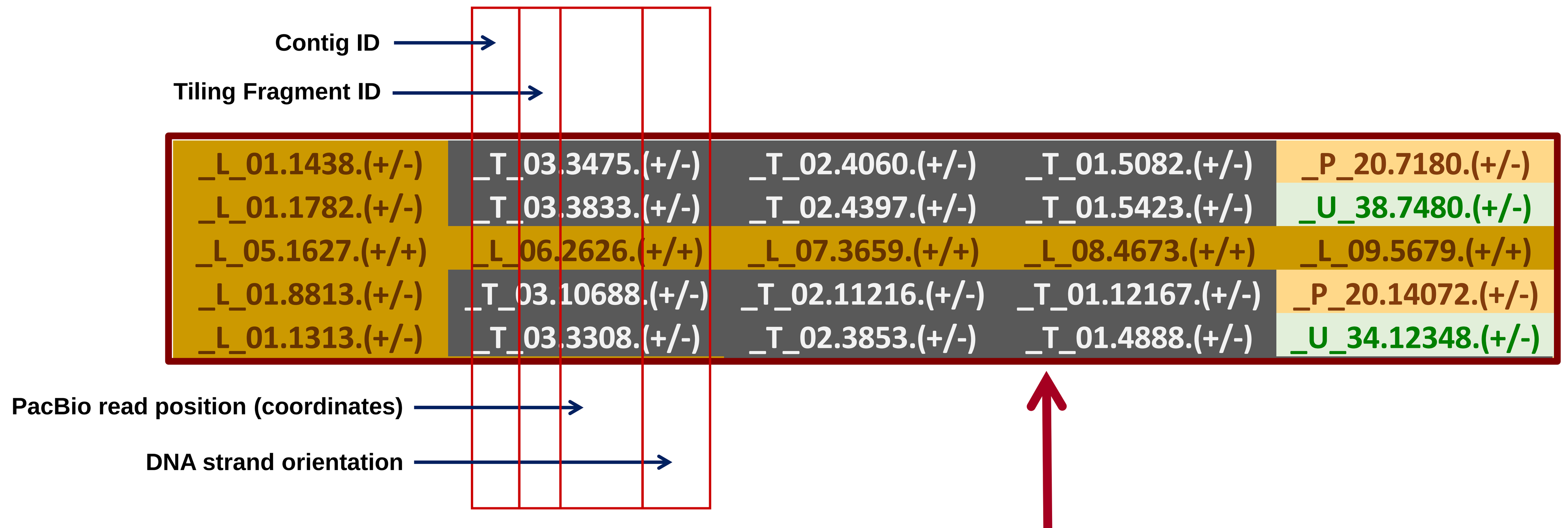

PacBio read ID										
m160218_092413_s1_p0:78128:0_20131	_L_03.11.(+/+)	_L_06.2903.(+/+)	_L_07.4017.(+/+)	_L_08.5032.(+/+)	_L_09.6083.(+/+)	_L_10.7107.(+/+)	_L_11.8178.(+/+)	_L_12.9197.(+/+)	_L_13.10223.(+/+)	_L_14.11342.(+/+)
m160207_141640_s1_p0:41172:0_36718	_L_03.12.(+/-)	_L_02.357.(+/-)	_L_01.1374.(+/-)	_T_03.3339.(+/-)	_T_02.3895.(+/-)	_T_01.4882.(+/-)	_P_20.6876.(+/-)	_P_19.7414.(+/-)	_P_18.8415.(+/-)	_P_17.9384.(+/-)
m160312_060406_s1_p0:56957:903_24293	_L_03.1233.(+/+)	_L_04.1726.(+/+)	_L_05.2803.(+/+)	_L_06.3818.(+/+)	_L_07.4910.(+/+)	_L_08.5955.(+/+)	_L_09.6894.(+/+)	_L_10.7905.(+/+)	_L_11.8904.(+/+)	_L_12.9913.(+/+)
m160207_054441_s1_p0:29215:40857_57262	_L_03.1447.(+/-)	_L_02.1955.(+/-)	_L_01.2920.(+/-)	_T_02.5824.(+/-)	_T_01.6262.(+/-)	_P_20.8065.(+/-)	_P_19.8539.(+/-)	_P_18.9546.(+/-)	_P_17.11039.(+/-)	_P_16.11333.(+/-)
m160208_030131_s1_p0:82895:0_11789	_L_03.175.(+/+)	_L_04.1407.(+/+)	_L_05.2140.(+/+)	_L_06.3171.(+/+)	_L_07.4210.(+/+)	_L_08.5221.(+/+)	_L_09.6235.(+/+)	_L_10.7244.(+/+)	_L_11.8247.(+/+)	_L_12.9480.(+/+)
m160313_073508_s1_p0:131401:0_15324	_L_03.3571.(+/+)	_L_04.4397.(+/+)	_L_05.5411.(+/+)	_L_06.6438.(+/+)	_L_07.7473.(+/+)	_L_08.8503.(+/+)	_L_09.9531.(+/+)	_L_10.10554.(+/+)	_L_11.11561.(+/+)	_L_12.12591.(+/+)
m160130_072631_s1_p0:108277:0_26197	_L_03.40.(+/-)	_L_02.384.(+/-)	_L_01.1438.(+/-)	_T_03.3475.(+/-)	_T_02.4060.(+/-)	_T_01.5082.(+/-)	_P_20.7180.(+/-)	_P_19.7648.(+/-)	_P_18.8677.(+/-)	_P_17.9749.(+/-)
m160130_072631_s1_p0:123773:0_11943	_L_03.43.(+/-)	_L_02.762.(+/-)	_L_01.1782.(+/-)	_T_03.3833.(+/-)	_T_02.4397.(+/-)	_T_01.5423.(+/-)	_U_38.7480.(+/-)	_U_37.8140.(+/-)	_U_36.9202.(+/-)	_U_35.10363.(+/-)
m160315_104902_s1_p0:86177:0_10112	_L_03.515.(+/+)	_L_04.640.(+/+)	_L_05.1627.(+/+)	_L_06.2626.(+/+)	_L_07.3659.(+/+)	_L_08.4673.(+/+)	_L_09.5679.(+/+)	_L_10.6679.(+/+)	_L_11.7686.(+/+)	_L_12.8706.(+/+)
m160208_030131_s1_p0:131569:37238_62585	_L_03.6897.(+/-)	_L_02.7839.(+/-)	_L_01.8813.(+/-)	_T_03.10688.(+/-)	_T_02.11216.(+/-)	_T_01.12167.(+/-)	_P_20.14072.(+/-)	_P_19.14589.(+/-)	_P_18.15574.(+/-)	_P_17.16506.(+/-)
m160207_203908_s1_p0:60476:0_17856	_L_03.8.(+/-)	_L_02.351.(+/-)	_L_01.1313.(+/-)	_T_03.3308.(+/-)	_T_02.3853.(+/-)	_T_01.4888.(+/-)	_U_34.12348.(+/-)	_U_33.12374.(+/-)	_U_32.13474.(+/-)	_U_31.14518.(+/-)
m160313_011220_s1_p0:125446:1297_24490	_L_04.108.(+/-)	_L_03.859.(+/-)	_L_02.1875.(+/-)	_L_01.2958.(+/-)	_T_03.5045.(+/-)	_T_02.5633.(+/-)	_T_01.6703.(+/-)	_P_20.8939.(+/-)	_P_19.9351.(+/-)	_P_18.10423.(+/-)
m160219_223641_s1_p0:36226:0_29738	_L_04.134.(+/-)	_L_03.608.(+/-)	_L_02.1624.(+/-)	_L_01.2696.(+/-)	_T_03.4699.(+/-)	_T_02.5272.(+/-)	_T_01.6265.(+/-)	_P_20.8349.(+/-)	_P_19.8785.(+/-)	_P_18.10331.(+/-)
m160209_105439_s1_p0:144127:0_20721	_L_04.15.(+/+)	_L_05.395.(+/+)	_L_06.1405.(+/+)	_L_09.6558.(+/+)	_L_10.6868.(+/+)	_L_11.8503.(+/+)	_L_12.8997.(+/+)	_L_13.10006.(+/+)	_L_14.10994.(+/+)	_L_15.12069.(+/+)
m160208_092334_s1_p0:109972:5842_20525	_L_04.2305.(+/+)	_L_05.2612.(+/+)	_L_06.3639.(+/+)	_L_07.4613.(+/+)	_L_08.5589.(+/+)	_L_09.6600.(+/+)	_L_10.7595.(+/+)	_L_11.8632.(+/+)	_L_12.9695.(+/+)	_L_14.12119.(+/+)
m160130_072631_s1_p0:82576:0_20938	_L_04.2405.(+/+)	_L_05.3236.(+/+)	_L_06.4247.(+/+)	_L_07.5244.(+/+)	_L_08.6252.(+/+)	_L_09.7247.(+/+)	_L_10.8246.(+/+)	_L_11.9289.(+/+)	_L_12.10310.(+/+)	_L_13.11934.(+/+)
m160208_030131_s1_p0:56234:2484_24273	_L_04.2954.(+/+)	_L_05.3563.(+/+)	_L_06.4545.(+/+)	_L_07.5608.(+/+)	_L_08.6556.(+/+)	_L_11.11363.(+/+)	_L_12.12208.(+/+)	_L_13.13135.(+/+)	_L_14.14129.(+/+)	_L_15.15147.(+/+)
m160313_202303_s1_p0:72365:1147_14545	_L_04.299.(+/+)	_L_05.854.(+/+)	_L_06.1874.(+/+)	_L_07.2907.(+/+)	_L_08.4081.(+/+)	_L_09.5073.(+/+)	_L_10.6201.(+/+)	_L_11.7175.(+/+)	_L_12.8218.(+/+)	_L_13.9275.(+/+)
m160315_171504_s1_p0:5339:33500_52346	_L_04.4.(+/-)	_L_03.730.(+/-)	_L_02.1720.(+/-)	_L_01.2705.(+/-)	_T_03.4642.(+/-)	_T_02.5188.(+/-)	_T_01.6165.(+/-)	_U_38.8191.(+/-)	_U_37.8804.(+/-)	_U_36.9777.(+/-)
m160310_011616_s1_p0:56095:0_28493	_L_04.4247.(+/-)	_L_03.4404.(+/-)	_L_02.5461.(+/-)	_L_01.6547.(+/-)	_T_03.8519.(+/-)	_T_02.9083.(+/-)	_T_01.10086.(+/-)	_U_38.12100.(+/-)	_U_37.12772.(+/-)	_U_36.13788.(+/-)
m160315_042634_s1_p0:68367:0_28375	_L_04.440.(+/-)	_L_03.854.(+/-)	_L_02.1859.(+/-)	_L_01.2901.(+/-)	_T_03.4934.(+/-)	_T_02.5509.(+/-)	_T_01.6540.(+/-)	_P_20.8609.(+/-)	_P_19.9140.(+/-)	_P_18.10202.(+/-)
m160208_030131_s1_p0:66210:36300_52616	_L_05.1.(+/-)	_L_06.1.(+/-)	_L_04.963.(+/-)	_L_03.1933.(+/-)	_L_02.2878.(+/-)	_L_01.3855.(+/-)	_T_03.5803.(+/-)	_T_02.6312.(+/-)	_T_01.7329.(+/-)	_U_38.9258.(+/-)
m160314_111808_s1_p0:111813:0_20845	_L_05.1159.(+/+)	_L_06.2071.(+/+)	_L_07.3032.(+/+)	_L_08.4003.(+/+)	_L_09.4950.(+/+)	_L_10.5941.(+/+)	_L_11.6955.(+/+)	_L_12.7847.(+/+)	_L_13.8834.(+/+)	_L_14.9814.(+/+)
m160218_092413_s1_p0:96518:6518_23230	_L_05.13.(+/-)	_L_04.501.(+/-)	_L_03.1465.(+/-)	_L_02.2353.(+/-)	_L_01.3264.(+/-)	_T_03.5184.(+/-)	_T_02.5612.(+/-)	_T_01.6561.(+/-)	_U_38.8441.(+/-)	_U_37.9120.(+/-)
m160208_154539_s1_p0:129224:0_17712	_L_05.29.(+/-)	_L_04.850.(+/-)	_L_03.1772.(+/-)	_L_02.2797.(+/-)	_L_01.3825.(+/-)	_T_03.5814.(+/-)	_T_02.6363.(+/-)	_T_01.7379.(+/-)	_U_38.9373.(+/-)	_U_37.10038.(+/-)
m160208_220812_s1_p0:22834:0_24599	_L_05.37.(+/+)	_L_06.697.(+/+)	_L_07.1400.(+/+)	_L_08.2416.(+/+)	_L_09.3423.(+/+)	_L_10.4348.(+/+)	_L_11.5380.(+/+)	_L_12.6404.(+/+)	_L_13.7431.(+/+)	_L_14.8463.(+/+)

Parsed BLAST-N data with detailed info about positioning of tiling fragments over PacBio reads

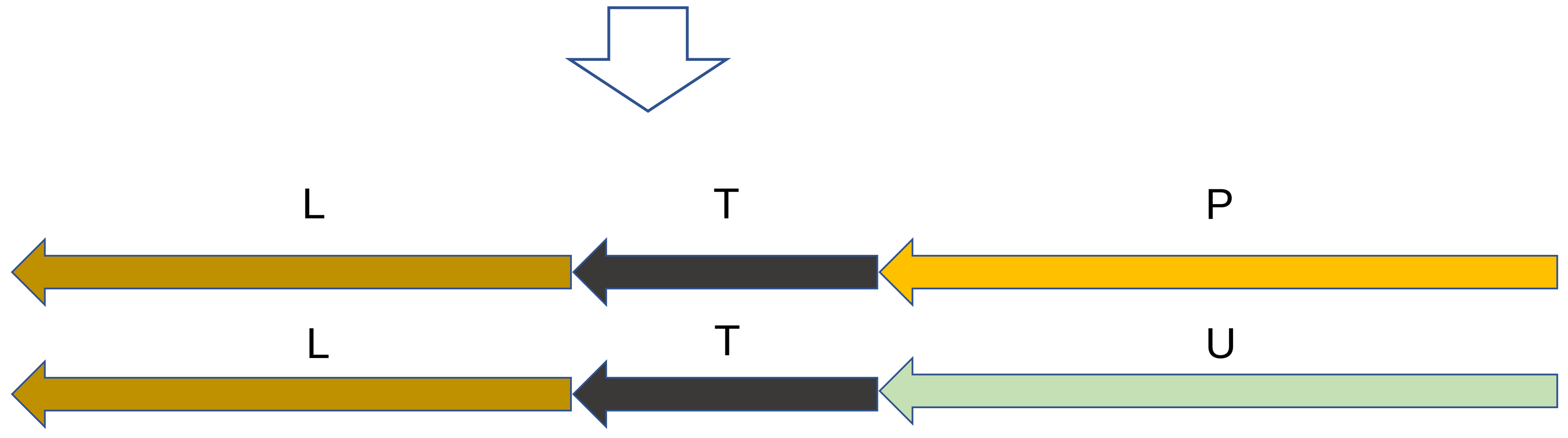

Inferred secondary building blocks

Reverse Read Mapping protocol outline

1. Construction of primary structural units (contigs) of mitochondrial genome with CLC PacBio assembler
2. Overlapping tiling library of 2 kb long segments for primary structural units (contigs)
3. Alignment/mapping of overlapping tiling library to raw PacBio reads (PacBio reads is a reference)
4. Analysis of sequential order of primary structural units over PacBio reads
5. Compilation of a library of secondary building blocks and inference of isoforms.

S9 Figure
