# Supplementary material for: The alternative reality of plant mitochondrial DNA: One ring does not rule them all
Source: PLoS Genet. 2019 Aug 30;15(8):e1008373. doi: 10.1371/journal.pgen.1008373 (PMC6742443; doi:10.1371/journal.pgen.1008373)
Supplement: S1 Table — A list of contemporary publications, many of which present a “master circle” model of the plant mitochondrial genome. The classifications reflect our best efforts to understand the data representation. (PDF) [file pgen.1008373.s010.pdf]

Publication	Year	DOI	Master Circle only/primary form	Acknowledged other forms	Other
Lin, C.P., et al., The complete mitochondrial genome of mungbean *Vigna radiata* var. *radiata* NM92 and a phylogenetic analysis of crops in angiosperms. Mitochondrial DNA A DNA Mapp Seq Anal, 2016. 27(5): p. 3731-2.	2016	10.3109/19401736.2015.1079879	X		
Duan, N., et al., The complete mitochondrial genome sequence of *Malus hupehensis* var. *pinyiensis*. Mitochondrial DNA A DNA Mapp Seq Anal, 2016. 27(4): p. 2905-6.	2016	10.3109/19401736.2015.1060432	X		
Kersten, B., et al., Genome Sequences of *Populus tremula* Chloroplast and Mitochondrion: Implications for Holistic Poplar Breeding. PLoS One. 2016 Jan 22;11(1):e0147209	2016	10.1371/journal.pone.0147209	X		
Guo, W., et al., *Ginkgo* and *Welwitschia* Mitogenomes Reveal Extreme Contrasts in Gymnosperm Mitochondrial Evolution. Mol Biol Evol, 2016. 33(6): p. 1448-60.	2016	10.1093/molbev/msw024	X		
Kim, B., et al., Completion of the mitochondrial genome sequence of onion (*Allium cepa* L.) containing the CMS-S male-sterile cytoplasm and identification of an independent event of the ccmF N gene split. Curr Genet, 2016. 62(4): p. 873-885.	2016	10.1007/s00294-016-0595-1	X	X	
Asaf, S., et al., Mitochondrial Genome Analysis of Wild Rice (*Oryza minuta*) and Its Comparison with Other Related Species. PLoS One, 2016. 11(4): p. e0152937.	2016	10.1371/journal.pone.0152937	X		
Yang, J., et al., Comparative mitochondrial genome analysis reveals the evolutionary rearrangement mechanism in *Brassica*. Plant Biol (Stuttg), 2016. 18(3): p. 527-36.	2016	10.1111/plb.12414	X		
Li, F., et al., The complete mitochondrial genome sequence of Sua-type cytoplasmic male sterility of tobacco (*Nicotiana tabacum*). Mitochondrial DNA A DNA Mapp Seq Anal, 2016. 27(4): p. 2929-30.	2016	10.3109/19401736.2015.1060445			X (linear)
Bellot, S., et al., Assembled Plastid and Mitochondrial Genomes, as well as Nuclear Genes, Place the Parasite Family Cynomoriaceae in the Saxifragales. Genome Biol Evol, 2016. 8(7): p. 2214-30.	2016	10.1093/gbe/evw147	X		
Kazama, T. and K. Toriyama, Whole Mitochondrial Genome Sequencing and Re-Examination of a Cytoplasmic Male Sterility-Associated Gene in Boro-Taichung-Type Cytoplasmic Male Sterile Rice. PLoS One, 2016. 11(7): p. e0159379.	2016	10.1371/journal.pone.0159379	X		
Gui, S., et al., The mitochondrial genome map of *Nelumbo nucifera* reveals ancient evolutionary features. Sci Rep, 2016. 6: p. 30158.	2016	10.1038/srep30158	X	X	
Shearman, J.R., et al., The two chromosomes of the mitochondrial genome of a sugarcane cultivar: assembly and recombination analysis using long PacBio reads. Sci Rep, 2016. 6: p. 31533.	2016	10.1038/srep31533	X (two circles)		

Aljohi, H.A., et al., Complete Sequence and Analysis of Coconut Palm (*Cocos nucifera*) Mitochondrial Genome. PLoS One, 2016. 11(10): p. e0163990.	2016	10.1371/journal.pone.0163990	X		
Hisano, H., et al., Mitochondrial genome sequences from wild and cultivated barley (*Hordeum vulgare*). BMC Genomics, 2016. 17(1): p. 824.	2016	10.1186/s12864-016-3159-3	X		
Bi, C., et al., Analysis of the Complete Mitochondrial Genome Sequence of the Diploid Cotton *Gossypium raimondii* by Comparative Genomics Approaches. Biomed Res Int, 2016. 2016: p. 5040598.	2016	10.1155/2016/5040598	X		
Chen, Z., et al., Entire nucleotide sequences of *Gossypium raimondii* and *G. arboreum* mitochondrial genomes revealed A-genome species as cytoplasmic donor of the allotetraploid species. Plant Biol (Stuttg), 2017. 19(3): p. 484-493.	2017	10.1111/plb.12536		X	
Skippington, E., et al., Comparative mitogenomics indicates respiratory competence in parasitic *Viscum* despite loss of complex I and extreme sequence divergence, and reveals horizontal gene transfer and remarkable variation in genome size. BMC Plant Biol, 2017. 17(1): p. 49.	2017	10.1186/s12870-017-0992-8	X		
Tong, W., Q. He, and Y.J. Park, Genetic variation architecture of mitochondrial genome reveals the differentiation in Korean landrace and weedy rice. Sci Rep, 2017. 7: p. 43327.	2017	10.1038/srep43327	X		
Petersen, G., et al., Mitochondrial genome evolution in Alismatales: Size reduction and extensive loss of ribosomal protein genes. PLoS One, 2017. 12(5): p. e0177606.	2017	10.1371/journal.pone.0177606	X	X	X (one circular and one linear)
Silva, S.R., et al., The mitochondrial genome of the terrestrial carnivorous plant *Utricularia reniformis* (Lentibulariaceae): Structure, comparative analysis and evolutionary landmarks. PLoS One, 2017. 12(7): p. e0180484.	2017	10.1371/journal.pone.0180484	X	X	
Chen, Z., et al., Rapid evolutionary divergence of diploid and allotetraploid *Gossypium* mitochondrial genomes. BMC Genomics, 2017. 18(1): p. 876.	2017	10.1186/s12864-017-4282-5	X	X	
Sang-Ho Kang, et al., The multipartite mitochondrial genome of *Cynanchum wilfordii* (Gentianales: Apocynaceae) Mitochondrial DNA Part B, 2:2, 720-721	2017	10.1080/23802359.2017.1390405	X		
Yang, K., et al., Whole-genome sequencing of *Brassica oleracea* var. *capitata* reveals new diversity of the mitogenome. PLoS One, 2018. 13(3): p. e0194356.	2018	10.1371/journal.pone.0194356	X		

Lee, H.O., et al., Assembly of the Mitochondrial Genome in the Campanulaceae Family Using Illumina Low-Coverage Sequencing. Genes (Basel), 2018. 9(8).	2018	10.3390/genes9080383	X		
Dong, S., et al., The complete mitochondrial genome of the early flowering plant *Nymphaea colorata* is highly repetitive with low recombination. BMC Genomics, 2018. 19(1): p. 614.	2018	10.1186/s12864-018-4991-4	X		
Shi Y., et al., Assembly and comparative analysis of the complete mitochondrial genome sequence of *Sophora japonica* 'JinhuaiJ2.' PLoS One. 2018 Aug 16;13(8):e0202485	2018	10.1371/journal.pone.0202485	X		
Arimura, S.I., et al., The mitochondrial genome of an asymmetrically cell-fused rapeseed, *Brassica napus*, containing a radish-derived cytoplasmic male sterility-associated gene. Genes Genet Syst, 2018. 93(4): p. 143-148.	2018	10.1266/ggs.18-00005	X		
Kovar, L., et al., PacBio-Based Mitochondrial Genome Assembly of *Leucaena trichandra* (Leguminosae) and an Intrageneric Assessment of Mitochondrial RNA Editing. Genome Biol Evol, 2018. 10(9): p. 2501-2517.	2018	10.1093/gbe/evy179	X	X	
Liao, X., et al., Complete sequence of kenaf (*Hibiscus cannabinus*) mitochondrial genome and comparative analysis with the mitochondrial genomes of other plants. Sci Rep, 2018. 8(1): p. 12714.	2018	10.1038/s41598-018-30297-w	X		
Kim, H.T. and J.M. Lee, Organellar genome analysis reveals endosymbiotic gene transfers in tomato. PLoS One, 2018. 13(9): p. e0202279.	2018	10.1371/journal.pone.0202279	X	X	
Cole, L.W., et al., High and Variable Rates of Repeat-Mediated Mitochondrial Genome Rearrangement in a Genus of Plants. Mol Biol Evol, 2018. 35(11): p. 2773-2785.	2018	10.1093/molbev/msy176		X	
Wu, Z. and D.B. Sloan, Recombination and intraspecific polymorphism for the presence and absence of entire chromosomes in mitochondrial genomes. Heredity (Edinb), 2018.	2018	10.1038/s41437-018-0153-3	X	X	
Li, S., et al., The comparison of four mitochondrial genomes reveals cytoplasmic male sterility candidate genes in cotton. BMC Genomics, 2018. 19(1): p. 775.	2018	10.1186/s12864-018-5122-y	X		
Wang, S., et al., Assembly of a Complete Mitogenome of *Chrysanthemum nankingense* Using Oxford Nanopore Long Reads and the Diversity and Evolution of Asteraceae Mitogenomes. Genes (Basel), 2018. 9(11).	2018	10.3390/genes9110547	X		
Li, J., et al., The complete mitochondrial genome sequence of *Boechera stricta*. Mitochondrial DNA Part B, 3(2): p. 896-897.	2018	10.1080/23802359.2018.1501323	X		

Yu, X., et al., The complete mitochondrial genome of *Schisandra sphenanthera* (Schisandraceae). Mitochondrial DNA Part B, 2018. 3(2): p. 1246-1247.	2018	10.1080/23802359.2018.1532346			X (no mention of structure)
Tsujimura, Mai, and Toru Terachi. 2018. "Cytoplasmic Genome." In The *Allium* Genomes, edited by Masayoshi Shigyo, Anil Khar, and Mostafa Abdelrahman, 89–98. Cham: Springer International Publishing.	2018	10.1007/978-3-319-95825-5_6	X	X	
Wang, Xuelin, et al., 2018. Organellar Genome Assembly Methods and Comparative Analysis of Horticultural Plants. Horticulture Research 5 (January): 3.	2018	10.1038/s41438-017-0002-1	X		
Tsujimura M., et al., Multichromosomal structure of the onion mitochondrial genome and a transcript analysis. Mitochondrion. 2019 May 46:179-186.	2019	10.1016/j.mito.2018.05.001	X		
Sanchez-Puerta, MV, et al., Genome-scale transfer of mitochondrial DNA from legume hosts to the holoparasite *Lophophytum mirabile* (Balanophoraceae). Mol Phylogenet Evol. 2019 Mar;132:243-250	2019	10.1016/j.ympev.2018.12.006		X	
Brenner, WG, et al., High Level of Conservation of Mitochondrial RNA Editing Sites Among Four *Populus* Species. G3 (Bethesda). 2019 Mar 7;9(3):709-717	2019	10.1534/g3.118.200763	X		
Maughan PJ, et al., Mitochondrial and chloroplast genomes provide insights into the evolutionary origins of quinoa (*Chenopodium quinoa* Willd.). Sci Rep. 2019 Jan 17;9(1):185	2019	10.1038/s41598-018-36693-6	X		
Shen, Jia, et al., Rare Maternal and Biparental Transmission of the Cucumber Mitochondrial DNA Reveals Sorting of Polymorphisms among Progenies. TAG. Theoretical and Applied Genetics. Theoretische Und Angewandte Genetik 132 (4): 1223–33.	2019	10.1007/s00122-018-03274-0	X		
Pinard, D., A.A. Myburg, and E. Mizrachi, The plastid and mitochondrial genomes of *Eucalyptus grandis*. BMC Genomics, 2019. 20(1): p. 132.	2019	10.1186/s12864-019-5444-4		X	
Wang, S., et al., Evolution and Diversification of Kiwifruit Mitogenomes through Extensive Whole-Genome Rearrangement and Mosaic Loss of Intergenic Sequences in a Highly Variable Region. Genome Biol Evol, 2019. 11(4): p. 1192-1206.	2019	10.1093/gbe/evz063	X	X	
Rawal, H.C., et al., Decoding and analysis of organelle genomes of Indian tea (*Camellia assamica*) for phylogenetic confirmation. Genomics, 2019.	2019	10.1016/j.ygeno.2019.04.018	X	X	
Wu, Z., et al., Mitochondrial genome and transcriptome analysis of five alloplasmic male-sterile lines in *Brassica juncea*. BMC Genomics, 2019. 20(1): p. 348.	2019	10.1186/s12864-019-5721-2	X	X	
Gandini, C.L., et al., The complete organelle genomes of *Physochlaina orientalis*: Insights into short sequence repeats across seed plant mitochondrial genomes. Mol Phylogenet Evol, 2019. 137: p. 274-284.	2019	10.1016/j.ympev.2019.05.012		X	
