# Supplementary material for: The alternative reality of plant mitochondrial DNA: One ring does not rule them all
Source: PLoS Genet. 2019 Aug 30;15(8):e1008373. doi: 10.1371/journal.pgen.1008373 (PMC6742443; doi:10.1371/journal.pgen.1008373)
Supplement: S2 Table — Mitochondrial genome coordinates of primary structural units and recombinogenic medium size repeats on representative isoforms of corresponding GenBank files MK642355 (S2A), MK820672 (S2B), and MK759657 (S2C). (PDF) [file pgen.1008373.s011.pdf]

Mitochondrial genome coordinates of primary structural units on representative GenBank files

S2A Table

Primary structural units	Recombinogenic medium size repeats	Length	Orientation	*L.sativa* MK642355 (isoform A)	
				Start	End
N (copy #1)		4116	R	1	4116
	X-01b (copy #1)	576	R	3541	4116
M (copy #1)		30580	R	4117	34696
K		78272	R	34697	112968
N (copy #2)		4116	R	112969	117084
	X-01b (copy #2)	576	R	116509	117084
M (copy #2)		30580	R	117085	147664
Q		11283	R	147665	158947
T (copy #1)		3552	R	158948	162499
P		20468	R	162500	182967
R (copy #1)		10430	R	182968	193397
L		44054	R	193398	237451
T (copy #2)		3552	R	237452	241003
U		38613	R	241004	279616
R (copy #2)		10430	R	279617	290046
Z		19295	R	290047	309341
	X-01b (copy #3)	576	F	309342	309917
W		53983	R	309342	363324

S2B Table

Primary structural units	Recombinogenic medium size repeats	Length	Orientation	*L.serriola* MK820672 (isoform B)	
				Start	End
N (copy #1)		4116	R	1	4116
	X-01b (copy #1)	576	R	3541	4116
M (copy #1)		30580	R	4117	34696
K		78272	R	34697	112968
N (copy #2)		4116	R	112969	117084
	X-01b (copy #1)	576	R	116509	117084
M (copy #2)		30580	R	117085	147664
Q		11283	R	147665	158947
T (copy #1)		3552	R	158948	162499
U		38617	R	162500	201116
R (copy #1)		10430	R	201117	211546
L		44054	R	211547	255600
T (copy #2)		3552	R	255601	259152
P		20468	R	259153	279620
R (copy #2)		10430	R	279621	290050
Z		19295	R	290051	309345
	X-01b (copy #1)	576	F	309346	309921
W		53983	R	309346	363328

S2C Table

Primary structural units	Recombinogenic medium size repeats	Length	Orientation	*L.saligna* MK759657	
				Start	End
M (copy #1)		30534	R	1	30534
K		79109	R	30535	109643
T (copy #1)		4048	F	109644	113691
L		43583	F	113692	157274
R (copy #1)		10433	F	157275	167707
P		20420	F	167708	188127
T (copy #2)		4048	F	188128	192175
Q		11083	F	192176	203258
M (copy #2)		30534	F	203259	233792
	X-01a (copy #1)	877	F	233793	234669
UV		90023	R	233793	323815
R (copy #2)		10433	R	323816	334248
Z		19278	R	334249	353526
	X-01a (copy #2)	877	F	353527	354403
	S-01-IR (copy #1)	1218	F	353527	354744
S		14743	R	353527	368269
	S-01-IR (copy #2)	1218	R	367052	368269
	X-01a (copy #3)	877	R	367393	368269
